# Successful treatment of locally advanced lung cancer using late concurrent chemoradiation therapy administered after immune checkpoint inhibitor plus platinum chemotherapy

**DOI:** 10.1111/1759-7714.14200

**Published:** 2021-10-24

**Authors:** Taichi Matsubara, Shinkichi Takamori, Takatoshi Fujishita, Ryo Toyozawa, Kensaku Ito, Masafumi Yamaguchi, Takashi Seto, Tatsuro Okamoto

**Affiliations:** ^1^ Department of Thoracic Oncology National Hospital Organization Kyushu Cancer Center Fukuoka Japan

**Keywords:** chemoradiation therapy, ICI, locally non‐small cell lung cancer, small cell lung cancer

## Abstract

Concurrent chemoradiation therapy (CRT) is the standard of care for patients with unresectable stage II/III lung cancer. However, systemic chemotherapy is required for patients who are ineligible for radical radiation therapy. There is little evidence to date for the safety and efficacy of CRT administered after treatment with immune checkpoint inhibitors (ICIs). The cases reported here had inoperable stage III lung cancer (non‐small cell lung cancer and small cell lung cancer) and were ineligible for radical radiation therapy. They were administered ICIs plus chemotherapy and subsequently underwent late concurrent CRT. Because of the remarkable tumor shrinkage achieved by the ICIs plus chemotherapy, adverse events of CRT were tolerable. They were alive without tumor progression as of this report, over 1 year after CRT was terminated. CRT is administered with curative intent, while the intent of immunochemotherapy is palliative. Late concurrent CRT after immunochemotherapy is probably effective and tolerable. After treatment with systemic chemotherapy in patients judged ineligible for radical radiation therapy, radiation therapy should be reconsidered because of its importance once tumor shrinkage has been achieved.

## Graphical abstract

Late concurrent chemoradiation therapy following systemic therapy (immunotherapy or immunochemotherapy) should be considered as an important treatment option for patients initially judged ineligible for definitive chemoradiation therapy.

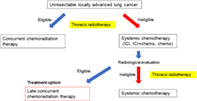

## INTRODUCTION

Chemotherapy combined with thoracic radiotherapy improves the prognosis of patients with non‐small cell lung cancer (NSCLC) and small cell lung cancer (SCLC).^1^, ^2^, ^3^, ^4^ This strategy is therefore the standard of care for unresectable and locally advanced cases during the early to middle stages. Furthermore, the PACIFIC trial found that maintenance administration of durvalumab after concurrent chemoradiotherapy (CRT) achieves favorable outcomes, thereby establishing durvalumab consolidation as a new worldwide standard of care for NSCLC.^5^ However, systemic chemotherapy combined with immune checkpoint inhibitors (ICIs) or cytotoxic chemotherapy is required for patients who are judged ineligible for radical radiation therapy.

ICIs are currently the foremost treatment strategy for patients with advanced lung cancer. Notably, combining ICIs and platinum‐based chemotherapy achieves favorable tumor responses and prognosis compared with standard chemotherapy administered to patients with advanced NSCLC and SCLC.^6^, ^7^, ^8^ Here, we report the cases of two patients who were judged ineligible for radical radiotherapy upon diagnosis and were therefore treated with ICIs plus platinum‐based chemotherapy followed by late concurrent CRT subsequent to shrinkage of the tumor.

## CASE REPORT

**FIGURE 1:**
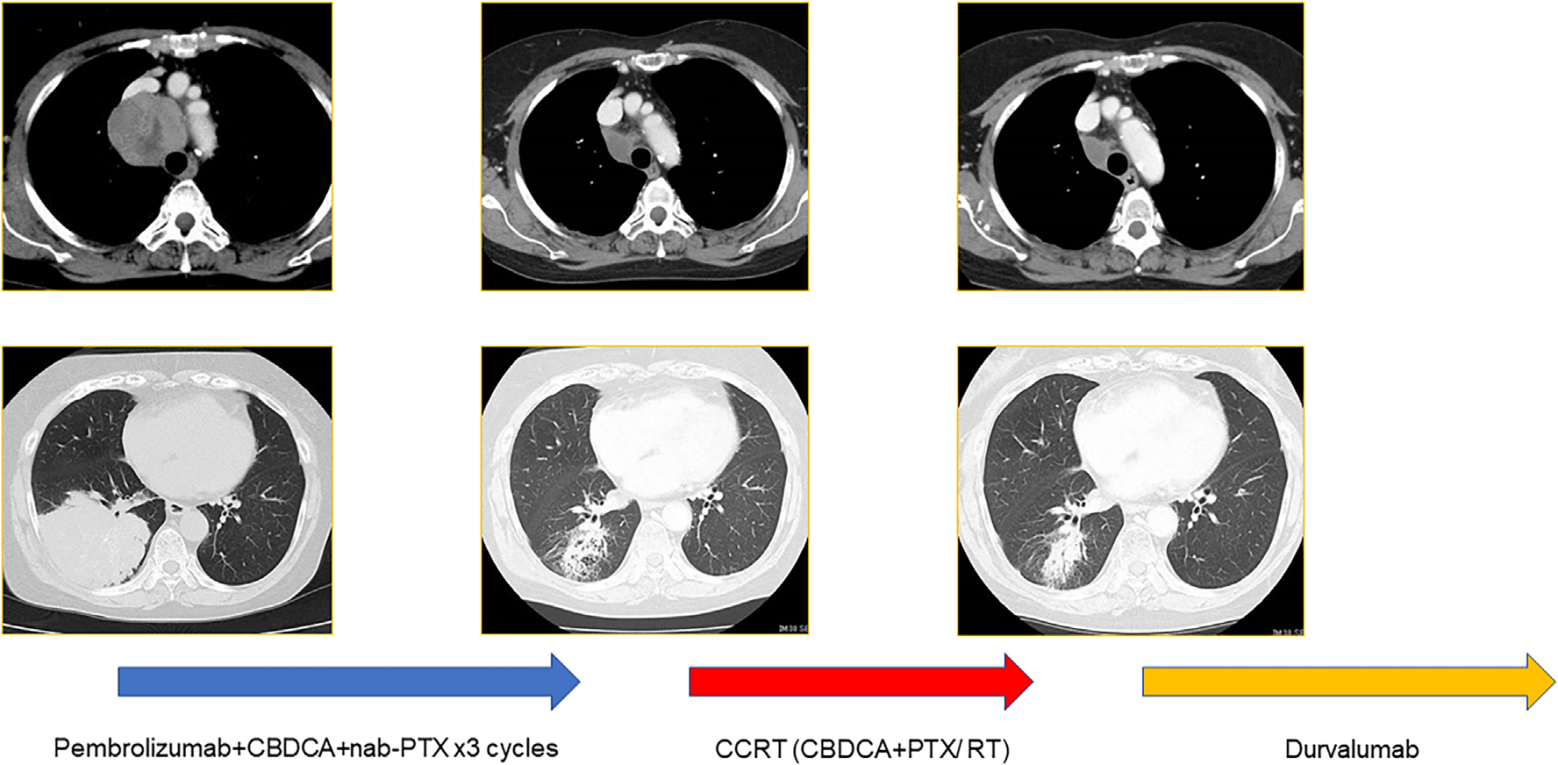
The first case involved a patient with inoperable stage III NSCLC who underwent CRT (CBDCA+PTX/60 Gy) following pembrolizumab + CBDCA + nab‐PTX. Durvalumab maintenance therapy was administered after CRT

The first patient was a woman in her 60s who was diagnosed with stage IIIB NSCLC (cT4N2M0 according to the eighth edition of the TNM classification).^9^ The primary tumor was >8 cm with hilar and mediastinal lymph node metastases detected using enhanced chest computed tomography (CT) and positron emission tomography (PET). Genetic testing showed no druggable alterations. The tumor proportion score for programmed death‐ligand 1 expression was <1%. Although radical irradiation was considered, we decided against it because the estimated lung V20 was >35%. Instead, treatment with pembrolizumab plus platinum‐based chemotherapy (carboplatin [CBDCA]) plus nab‐paclitaxel (nab‐PTX) was initiated. The primary site and lymph nodes underwent remarkable shrinkage after three cycles of this immunochemotherapy, and the recalculated lung V20 was 34.2%. Given this satisfactory shrinkage, we sequentially performed definitive concurrent CRT (CBDCA + PTX 2 cycles/RT [60 Gy/30 Fr]). Adverse events (AEs) during CRT comprised only grade 2 neutropenia as a hematological AE, and this was tolerable. After CRT, maintenance using durvalumab was initiated, and the patient completed 1 year of maintenance therapy without disease progression (Figure 1).

The second patient was a man in his 60s who visited our hospital because of a chronic cough and mild respiratory difficulty. He was diagnosed with SCLC on the basis of histological analysis of a transbronchial biopsy. Enhanced CT and PET revealed a primary tumor in the right upper lobe and showed multiple lymph node metastases in the right mediastinum and right hilum. Pleural effusion was also noted. A cytological examination did not find malignancy in the pleural effusion, and he was therefore diagnosed with limited‐stage SCLC.

**FIGURE 2:**
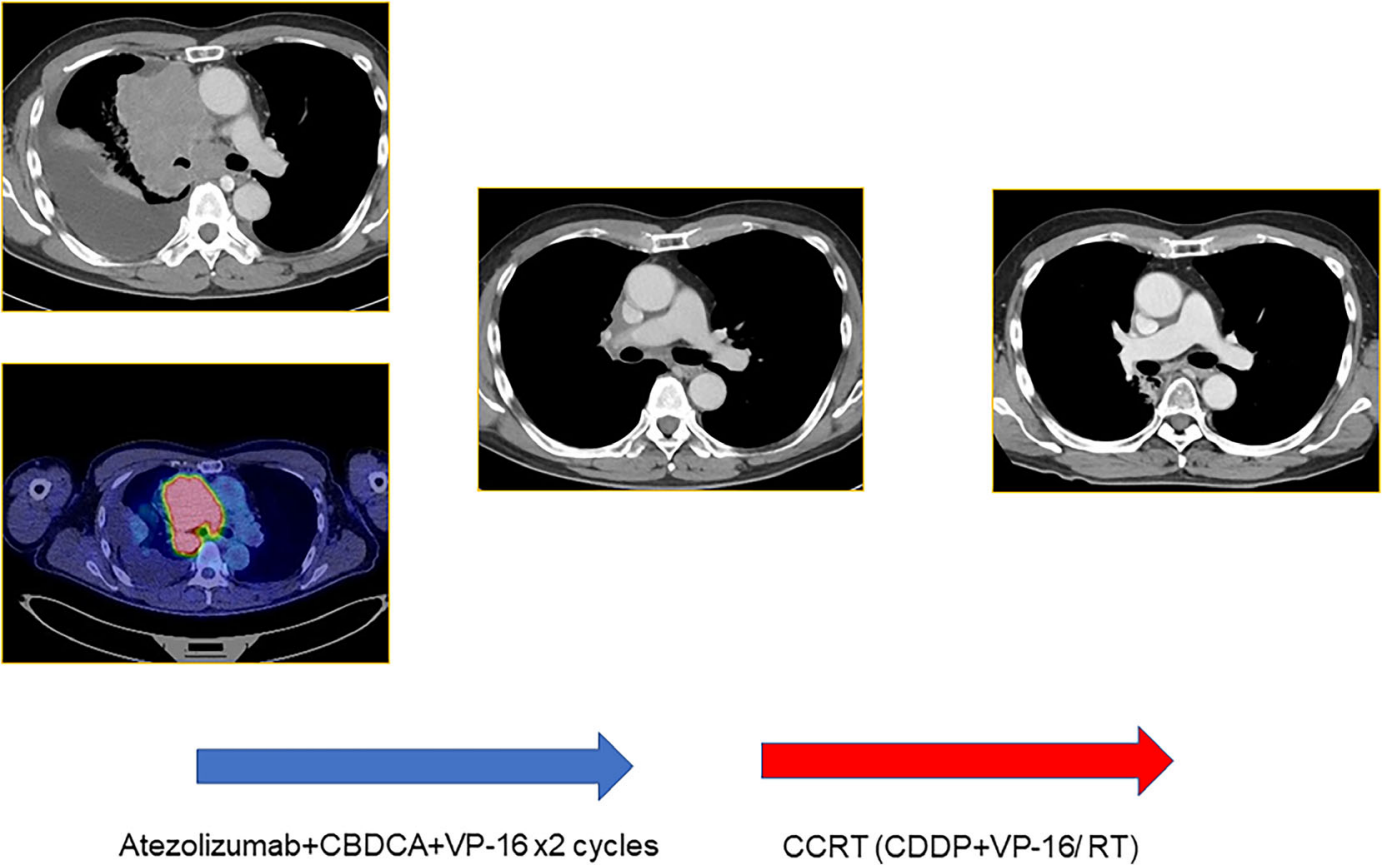
The second case involved a patient with stage III LS‐SCLC who was ineligible for concurrent CRT upon diagnosis. Tumor shrinkage was achieved during prior immunochemotherapy (atezolizumab + CBDCA+ETP), and subsequent CRT (CDDP + ETP/45 Gy)

The lung V20 in the radical radiation field was >35% (V20 was approximately 50%), and the patient was treated with systemic immunochemotherapy (atezolizumab + CBDCA + etoposide [ETP]). After two cycles of immunochemotherapy, marked shrinkage of the tumor was observed on CT, including the primary site and lymph nodes, which involved 15.0% of the lung V20. After completion of immunochemotherapy, the patient underwent concurrent CRT (cisplatin + ETP, two cycles/45 Gy) (Figure 2). AEs during CRT were neutropenia, nausea, and esophagitis, all of which were mild (grade 2). At the time of writing this report, the patient has remained alive without tumor progression for over 1 year after completion of CRT.

## DISCUSSION

Here, we report the cases of two patients who received late concurrent CRT subsequent to ICI plus platinum‐based chemotherapy for locally advanced NSCLC and SCLC. Remarkable tumor volume shrinkage in each case was induced with immunochemotherapy before CRT, allowing both patients to undergo successful radical radiation therapy with tolerable AEs.

The preferred treatment for locally advanced NSCLC or stage III SCLC combines chemotherapy with radical radiotherapy. Although concurrent CRT should be considered first, the Royal College of Radiologists recommends sequential chemotherapy and radical radiotherapy as an appropriate treatment option if the patient is insufficiently fit to undergo concurrent CRT. Therefore, even if systemic chemotherapy is introduced, definitive radiotherapy should be considered when the tumor shrinks during treatment.

Currently, ICI plus chemotherapy achieves a higher antitumor response compared with conventional standard chemotherapy,^7^, ^8^ which we anticipate will lead to an increase in the number of patients who become eligible for this optional CRT. Although immunochemotherapy (ICI plus chemotherapy) is a standard treatment for patients with locally advanced NSCLC who are ineligible for radical radiotherapy, we consider late concurrent CRT to be a suitable option once the patient becomes eligible for radical radiotherapy following systemic chemotherapy. The results of two recent phase 3 trials comparing the timing (early or late) of chest radiotherapy administered to patients with LS‐SCLC,^10^, ^11^ found no significant survival differences between early and late treatment. Furthermore, the rate of febrile neutropenia in the late arm was significantly lower in one study,^11^ and neither study found a significant difference between the two arms regarding other side effects.^10^, ^11^ In our two present cases, despite undergoing CRT subsequent to immunotherapy, the patients did not experience severe AEs, including hematological AEs and immune‐related AEs.

Here, we report two cases of patients who underwent late concurrent CRT after ICI plus chemotherapy, with a good safety profile. These findings highlight an important treatment option for patients initially judged ineligible for definitive CRT.

## CONFLICT OF INTEREST

S.T. received honoraria from AstraZeneca, Chugai Pharmaceutical, and Taiho Pharmaceutical. T.F. received honoraria from Taiho Pharmaceutical. R.T. received grants from Abbvie, Amgen, Daiichi Sankyo, Pfizer Japan, Takeda Pharmaceutical, Eli Lilly Japan, and Novartis Pharma, and honoraria from Bristol‐Myers Squibb, Chugai Pharmaceutical, Eli Lilly Japan, Kyowa Hakko Kirin, MSD, Nippon Boehringer Ingelheim, Nippon Kayaku, Novartis Pharma, and Taiho Pharmaceutical. M.Y. received grants from Chugai Pharmaceutical, Daiichi Sankyo, MSD, and Pfizer Japan, and honoraria from AstraZeneca, Chugai Pharmaceutical, Covidien Japan, Nippon Boehringer Ingelheim, Ono Pharmaceutical, and Taiho Pharmaceutical. T.S. received grants from Abbvie, Chugai Pharmaceutical, Daiichi Sankyo, Eli Lilly Japan, Kissei Pharmaceutical, Merck Biopharma, MSD, Novartis Pharma, Pfizer Japan, and Takeda Pharmaceutical, and honoraria from AstraZeneca, Bristol‐Myers Squibb, Chugai Pharmaceutical, Covidien Japan, Daiichi Sankyo, Eli Lilly Japan, Kyowa Hakko Kirin, MSD, Mochida Pharmaceutical, Nippon Boehringer Ingelheim, Novartis Pharma, Ono Pharmaceutical, Pfizer Japan, Taiho Pharmaceutical, Takeda Pharmaceutical, and Thermo Fisher Scientific, as well as salary compensation from Precision Medicine Asia. T.O. received grants from Bristol‐Myers Squibb, Chugai Pharmaceutical, Covidien Japan, Pfizer Japan, AstraZeneca, MSD, Nippon Boehringer Ingelheim, and Taiho Pharmaceutical, and honoraria from Eli Lilly Japan, Johnson & Johnson, AstraZeneca, MSD, Nippon Boehringer Ingelheim, and Taiho Pharmaceutical. The other authors have stated that they have no conflict of interest.
